# Time budgets and weight shifting as indicators of pain in hospitalized horses

**DOI:** 10.3389/fpain.2024.1410302

**Published:** 2024-07-23

**Authors:** Magdalena Nowak, Albert Martin-Cirera, Florien Jenner, Ulrike Auer

**Affiliations:** ^1^ Anesthesiology and Perioperative Intensive - Care Medicine Unit, Department of Companion Animals and Horses, University of Veterinary Medicine Vienna, Vienna, Austria; ^2^ Precision Livestock Farming Hub and Institute of Animal Husbandry and Animal Welfare, University of Veterinary Medicine Vienna, Vienna, Austria; ^3^ Equine Surgery Unit, Department of Companion Animals and Horses, University Equine Hospital, University of Veterinary Medicine Vienna, Vienna, Austria

**Keywords:** equine pain, equine discomfort, pain score, posture, time budgets, weight shifting

## Abstract

**Introduction:**

Pain assessment in horses presents a significant challenge due to their nonverbal nature and their tendency to conceal signs of discomfort in the presence of potential threats, including humans. Therefore, this study aimed to identify pain-associated behaviors amenable to automated AI-based detection in video recordings. Additionally, it sought to determine correlations between pain intensity and behavioral and postural parameters by analyzing factors such as time budgets, weight shifting, and unstable resting. The ultimate goal is to facilitate the development of AI-based quantitative tools for pain assessment in horses.

**Materials and methods:**

A cohort of 20 horses (mean age 15 ± 8) admitted to a university equine hospital underwent 24-h video recording. Behaviors were manually scored and retrospectively analyzed using Loopy® software. Three pain groups were established based on the Pain Score Vetmeduni Vienna : pain-free (P0), mild to moderate pain (P1), and severe pain (P2).

**Results:**

Weight shifting emerged as a reliable indicator for discriminating between painful and pain-free horses, with significant differences observed between pain groups (*p* < 0.001) and before and after administration of analgesia. Additionally, severely painful horses (P2 group) exhibited lower frequencies of feeding and resting standing per hour compared to pain-free horses, while displaying a higher frequency of unstable resting per hour.

**Discussion:**

The significant differences observed in these parameters between pain groups offer promising prospects for AI-based analysis and automated pain assessment in equine medicine. Further investigation is imperative to establish precise thresholds. Leveraging such technology has the potential to enable more effective pain detection and management in horses, ultimately enhancing welfare and informing clinical decision-making in equine medicine.

## 1 Introduction

Pain is a critical determinant of patient welfare and plays a crucial role in guiding clinical decisions. In horses, a nonverbal prey species inherently inclined to display minimal signs of pain in the presence of potential threats including humans (1–3), the assessment of pain poses a notorious challenge. Particularly, mild to moderate pain, whether acute or chronic, may lead to falsely low scores, resulting in an underestimation of pain intensity (4). Physiologic parameters like heart and respiratory rate lack the requisite sensitivity and specificity for reliable pain detection and differentiation from other sources of distress (5–7). Consequently, the focus has shifted toward investigating pain behaviors, such as facial expressions and alterations in activity patterns or mental status, as indicators of pain.

As healthy, stress-free horses adhere to highly repetitive, individual daily routines with specific time allocations for different activities (time budgets), deviations from these established time budgets can serve as signals of discomfort, pain, or potential disease (2, 3, 7–9). However, accurate time budget analysis requires continuous observation over extended periods, limiting its practicality for pain evaluation in clinical settings. Automated video analysis emerges as a promising solution, eliminating the need for continuous human observation and facilitating the use of time budgets for early pain and health issue detection.

Healthy horses evenly distribute their weight, with 60% of the weight on the forelimbs and 40% on the hind limbs (8). Although horses may occasionally rest one hind limb at a time, their overall weight distribution remains balanced (8). Horses suffering from orthopedic pain may reduce the load on the affected limb by positioning it away from the center of gravity e.g., by pointing the affected limb (9–11). Notably, postural adjustments, aimed at minimizing the load on painful tissues to prevent or alleviate pain and safeguard against further injury, exhibit a strong association with orthopaedic pain in both humans and horses (11). These postural adjustments lend themselves to automated video analysis, thus opening avenues for the development of a real-time, continuous, and objective quantification of pain. Despite these advancements, no study has yet established a definitive link between the degree of weight shifting and equine discomfort or pain.

Therefore, this study aims to identify pain-associated behaviors amenable to automated AI-based detection in video recordings and determine correlations between pain intensity and behavioral and postural parameters. We hypothesized that, time budgets, weight shifting, and unstable resting are potentially good parameters to identify equine pain.

## 2 Material and methods

### 2.1 Horses and video recording

Horses admitted to the University Equine Hospital of the University of Veterinary Medicine Vienna are allocated randomly to 4 × 4 m box stalls based on availability, with the stables being bedded with shavings and cleaned twice daily.

This study recruited a cohort of 200 horses assigned to one of four stables equipped with video surveillance cameras during the period spanning from April to November 2021, with the owner's consent. Inclusion criteria mandated hospitalization for a minimum of three consecutive days to allow for a period of acclimatization lasting at least 24 h post-admission before the onset of video recording. Horses were video recorded for 24-h employing either a GoPro® action camera or an Acaris webcam (Horse Protector®) camera. These cameras were strategically positioned at a height of 2.5 m in a corner at the front of the box stall, affording a panoramic view of the entire enclosure. The recordings were made in time lapse mode with two picture per second. Only horses that received full rations of food, comprising hay dispensed four times daily, were eligible for inclusion in the study. All horses had unlimited access to water.

From the initial pool of 200 horses, a subset of 20 animals was randomly chosen for analysis, irrespective of the cause for hospitalization and the pain status of the horses (refer to Table 1).

**Table 1:** Horses’ diagnosis, medication, and pain scores post-admission (postadm) and post-surgery (postsurg).

Horse	Breed	Sex	Age	Weight	Diagnosis	Day of video recording after surgery/admission	Pain medication	Pain group	Mean pain score
A	Miniature Warmblood	M	30	275	Chronic laminitis	No surgery, d2 postadm	Firocoxib 1x daily PO	P1	4
B	Warmblood	F	6	560	Tremor of unknown origin	No surgery, d2 postadm	Phenylbutazone 2× daily PO	P0	2
C	Coldblood	F	21	618	Abscess on lower breast	d3 postsurg	Phenylbutazone 2× daily PO	P1	3
D	Warmblood	M	13	636	Septic tarsocrural joint	d3 postsurg	Flunixin Meglumine 2× daily IV	P1	4
E	Warmblood	F	23	615	Lameness front limb grade 2/5	No surgery d2 postadm	Firocoxib 1× daily PO	P0	2
F	Warmblood	M	6	500	Old wound front limb	d3 postadm	Flunixin Meglumine 2× daily IV	P0	2
G	Warmblood	M	23	480	Equine asthma, cough	No surgery d2 postadm.	No therapy	P0	0
H	Warmblood	M	8	558	Osteoarthritis tarsus	No surgery d2 postadm	No therapy	P0	2
I	Warmblood	M	14	604	Septic nuchal bursa	d4 postsurg	No therapy	P1	7
J	Warmblood	M	2	340	Colic	d3 postsurg	Flunixin Meglumine 2× daily IV	P0	1
K	Warmblood	F	14	555	Colic	d6 postsurg	Flunixin Meglumine 1× daily PO	P1	4
L	Warmblood	M	19	451	Colic	d3 postsurg	Flunixin Meglumine 2× daily IV	P1	5
M	Coldblood	M	25	636	Lameness stifle	No surgery d2 postadm	No therapy	P1	4
N	Warmblood	F	10	550	Olecranon fracture	d19 postsurg	Phenylbutazone 2× daily PO	P1	3
O	Warmblood	F	13	530	Osteosynthesis P1 fracture	d3 postsurg	Phenylbutazone 2× daily PO	P1	5
P	Coldblood	F	15	770	Dental problem	No surgery d2 postadm	No therapy	P1	2
Q	Warmblood	M	26	480	Septic arthritis	d2 postsurg	Flunixin Meglumine 2× daily IV	P2	10
R	Warmblood	M	12	519	Choke	No surgery, d2 postadm	No therapy	P0	0
S	Warmblood	F	7	598	Epileptic episode	No surgery, d2 postadm	Flunixin Meglumine 2× daily IV	P0	0
T	Warmblood	F	3	348	Septic arthritis	d3 postsurg	Flunixin Meglumine 2× daily IV	P2	11

d, day; IV, intravenous, PO, per os. P0, pain free group; P1, group with mild to moderate pain; P2, group with severe pain.

The recording period commenced no earlier than the second day of admission when the first set of 24 videos was available after admission and no surgery. In the case of surgery, the full set of 24-h videos was available the day after surgery when the horses were on a full food ratio.

### 2.2 Pain medication, examination and pain score

**Figure 1:**
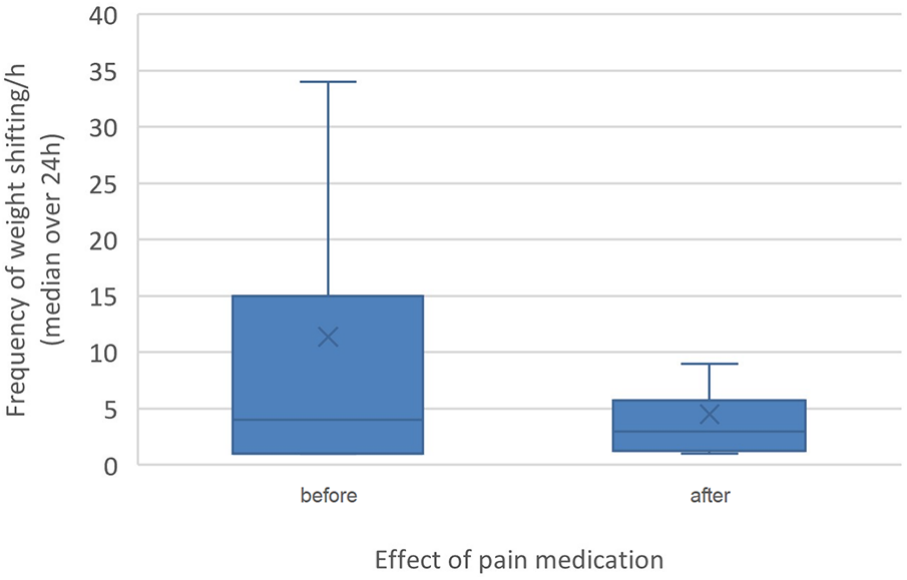
Effect of pain medication (before pain medication versus after) on weight shifting. The difference in weight shifting was statistically significant (*p* < 0.001).

During their hospital stay, all horses were examined at least twice daily, at 8:00 am and 8:00 pm, which included a comprehensive physical exam and determination of pain, using the Pain Score Vetmeduni Vienna (12), (Supplementary Files, Figure 1). Treatments, including the administration of pain medication as deemed necessary, were provided according to each horse's specific medical condition, determined solely by the clinician's discretion, and were not influenced by the study (Table 1). Exam and treatment data were collected retrospectively from the digital medical history.

Based on the mean pain score from two pain assessments conducted over 24 h, the horses were stratified *post hoc* into three groups: the pain-free group (P0, score ≤ 2), the mildly to moderately painful group (P1, 2 < score ≤ 8) or the severely painful group (P2 score > 8).

### 2.3 Behaviour scoring

Behaviors were systematically assessed using Loopy® (Loopbio, Vienna, Austria), a video coding interface that supports the coding of a wide range of behaviors for multiple individuals and provides corresponding plotting and analysis tools. Surveillance videos were uploaded into the software and time corresponding to the presence of veterinary professionals, nurses, technicians, or students in the stall during activities such as feeding, medication administration, or examination, was deducted from the total video duration. After an ethogram was defined in the program (Table 2), videos underwent manual behaviour scoring by a veterinarian (M.N.), who was blinded to the medical history and treatment of the patients and consistently adhered to the behavior-scoring guidelines adapted from V Boy and others (13) (Table 2).

**Table 2:** Ethogram used to manually score horses’ behavior.

Lying	The horse is lying in lateral or sternal recumbency; the duration of lying is measured from the moment the horse lies down to when it resumes a standing position.
Resting standing (RS)	The horse is motionless (not eating), asleep or drowsy, allowing the ears and tail to move, with the head held motionless at height of the withers or slightly above or below.
Unstable resting (UR)	The horse remains stationary (taking fewer than 3 steps in any direction, not eating), displaying small, restless movements or behaviors that are often repetitive and can include actions like shifting weight with or without lifting the limbs, swaying, nodding the head, or other subtle gestures, described as fidgeting by Torcivia and McDonell (3), either in a state of drowsiness or alertness while observing the surroundings.
Feeding and foraging (feed)	Activities such as eating, foraging, nibbling or sniffing food either on the ground or in a feeder, or actively searching for food. The onset of feeding behavior was marked from the moment the horse lowered its head and started to eat or forage until it raised the head again.
Locomotion	Forward or backward movement of more than one limb for more than three steps resulting in a new position within the stable.
Weight shifting (WS)	Scored as an event; Frequent shifting of the primary weight-bearing limb or limbs with or without lifting the hooves.

Initially, the assessment focused on resting standing, feeding, lying, and movement behaviors as well as weight shifting. The resting phase, defined by a lack of ambulation or eating, was later subdivided into unstable resting and resting standing, collectively referred to as total resting time. Unstable resting behavior was counted during the total resting phase if it persisted for more than 10 frames (=5 s).

### 2.4 Time budgets

Data from Loopy® were extracted as a CSV (Comma-Separated Values) file for subsequent analysis. The duration of five behavioral categories—feeding, resting standing, unstable resting, locomotion, and lying—was quantified per hour. The mean duration of each behavior episode within its respective category was computed per hour and labeled as duration of feeding (D_feed), resting standing (D_RS), and unstable resting (D_UR). Additionally, the total activity count, representing the number of behavior switches (not including weight shifting) per hour, and the frequency of occurrences of feeding (C_feed), resting (C_RS), and unstable resting (C_UR) were documented as means per hour over a 24-h period. The total resting time (TRT) was obtained by summing the durations of resting standing (RS) and unstable resting. Subsequently, time budgets were calculated as a percentage of time each horse spent on each behavior divided by the observation (video) time and then we obtained a mean per hour.

### 2.5 Weight shifting

Weight shifting (WS) was scored as an event and reported as number of events per hour. Additionally, the ratio of the number of weight shifts to the total resting time (WS/TRT) per hour was calculated to provide a relation to the resting time.

### 2.6 Statistical analysis

NCSS Statistical Software® [NCSS 2023 Statistical Software (2023). NCSS, LLC. Kaysville, Utah, USA, ncss.com/software/ncss.] was used for data analysis. Kolmogorov-Smirnov tests were performed on the data to assess normality. Data are presented as median and range (min-max). The impact of pain medication (yes/no), as well as before and after treatment, and the pain group on time budgets and weight shifting was assessed using Kruskal-Wallis tests. Subsequently, a *post hoc* analysis was conducted with the Kruskal-Wallis Multiple Comparison *Z*-Value Test (Dunn's Test), with statistical significance set at a *p*-value less than 0.05.

## 3 Results

### 3.1 Horses

The horses' mean age was 15 ± 8 years, and their mean weight was 534 ± 139 kg. Further details about the population, including medication specifics and reasons for admission, are provided in Table 2.

### 3.2 Time budget

The median time budget over 24 h for feeding was 46% (range: 0–97), for resting it was 16% (range: 0–82), and for unstable resting it was 19% (range: 2–96). Total resting comprised 47% (range: 2–96), while locomotion accounted for 0.5% (range: 0–36). Fourteen horses were observed lying during the 24-hour period, with a mean time budget of 4 ± 15%. The median frequency of weight shifts per hour was 3 (range: 1–106), while the total activity count was recorded as 24/h (range: 1–269). Detailed time budgets per horse are provided in (Supplementary Table S1).

### 3.3 Medication

Six (30%) horses received no pain medication (Table 1), two of which (K, V) were assigned to P0 and the other to P1 (J, L, P, S). Three horses (15%, one in P0, two in P1) received phenylbutazone (2 mg/kg, PO or IV BID), six horses (30%, two in P0, three in P1 and two in P2) flunixin meglumine (1,1 mg/kg, IV, BID) and two horses (10%, one in P0, one in P1) firocoxib (0,1 mg/kg, PO, SID).

**Figure 2:**
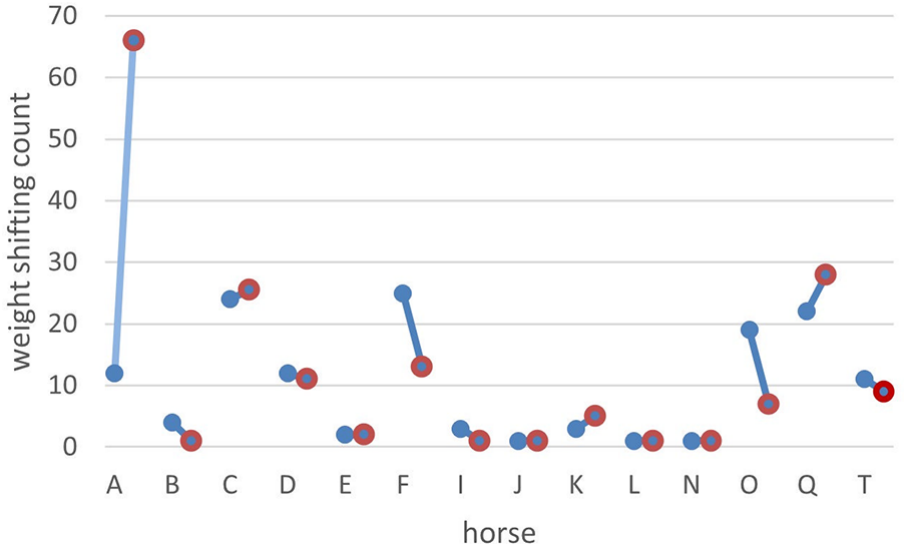
Effect of pain medication on weight shifting comparing the three hours before medication (blue dots) to the three hours after medication (red dots). On the x-axis are the horses (indicated by their ID), and on the y-axis is the count of weight shifting before and after treatment. Horse A was in the pain group P1, with the diagnosis chronic laminitis and received Firocoxib orally once per day.

Pain medication had no significant influence on the time budgets. The frequency of weight shifting per hour was significant lower (*p* < 0.001, median weight shifts/h = 2, range: 1–43) in horses without pain medication compared to the horses that received pain medication (median weight shifts/h = 5, range: 1–106). Although pain medication was not adjusted based on horses' pain score but rather administered based on clinician preference, horses showed significantly (*p* < 0.001) less weight shifting (median weight shifts/h = 3, range: 1–106) after receiving pain medication than before (median weight shifts/h = 4, range 1–56, Figures 1, 2). However, the response to treatment was individually variable.

### 3.4 Pain groups

Based on the Pain Score Vetmeduni Vienna, eight horses were allocated to P0, ten horses to P1 and two horses to P2.

**Figure 3:**
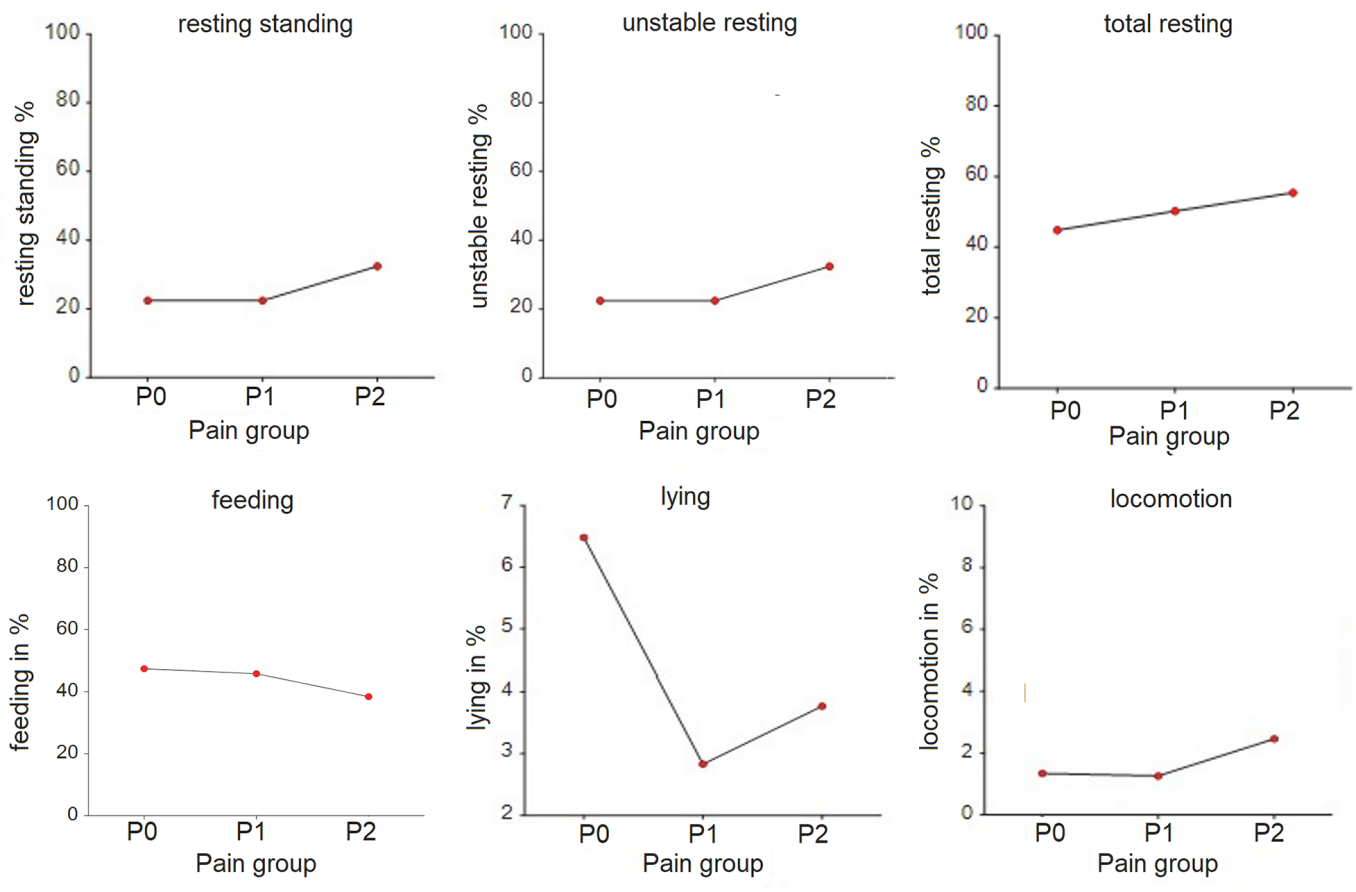
Time budgets (in %) for lying, feeding, locomotion, unstable resting, resting standing and total resting by pain group allocation of the horses. P0 – pain free group, *n* = 8; P1 – group with mild to moderate pain, *n* = 10; P2 – group with severe pain, *n* = 2. The grouping is done based on the Pain Score Vetmeduni Vienna.

The time budgets for lying (*p* < 0.001) and total rest (*p* = 0.011) were significantly different between pain groups (Table 3, Figure 3). The time budget for feeding was lower (*p* = 0.247), but the time budgets for resting standing (*p* = 0.141) and unstable resting (*p* = 0.44) were higher in P2 compared to P0 and P1.

**Table 3:** Discomfort indices and time budgets per pain group.

Parameter	P0	P1	P2	*p*-value
Weight shifting (per h)	2 (1–86)*	3 (1–106)	13 (1–67)	<0.001
Weight shifting/total resting time (WS/TRT)	0.05 (0–1.6)*	0.15 (0–3.4)	0.34 (0–1.8)	<0.001
Feeding (%)	48 (1–100)	46 (0–100)	34 (0–100)	0.247
Resting standing (%)	17 (1–89)	12 (0–99)	24 (0.89)	0.141
Unstable resting (%)	18 (1–94)	21 (0–100)	17 (0–100)	0.44
Locomotion (%)	0.5 (0–16)	0,3 (0–20)	0 (0–36)	0.05
Lying (%)	0 (0–100)	0 (0–99)	0 (0–79)	<0.001
Total resting (%)	43 (1–100)	49 (0–100)	64 (0–100)*	0.011
Total activity/h	23 (1–203)	25 (1–188)	8 (2–99)*	0.032
Frequency of feeding/h	5 (1–34)	5 (1–70)	2 (1–22)*	<0.003
Frequency of resting standing/h	4 (1–68)	4 (0,43–73)	2 (1–15)*	<0.001
Frequency of unstable resting/h	9 (1–50)	8 (1–47)	10,5 (1–48)*	0.001
Duration of feeding (min)/h	3 (9–47)	6 (0–107)*	4 (0–43)	0.014
Duration of unstable resting (min)/h	1 (0–28)	2 (0–32)	0.5 (0–8)*	<0.001
Duration of resting standing (min)/h	1.5 (0–21)	2 (0–57)	9 (0–87)*	<0.001

Values are provided as median and range by pain group based on the Pain Score Vetmeduni Vienna. P0 – pain free group; P1 – group with mild to moderate pain; P2 – group with severe pain. (*)- indicates statistical significance from other groups, with a *p*-value lower than 0.05.

The frequency of feeding (*p* = 0.003) and resting standing (*p* < 0.001) per hour was significantly lower in the P2 group compared to P1, while the frequency of unstable resting (*p* = 0.001) was significantly higher in the P2 group compared to P1. Additionally, the mean duration of resting standing was significantly longer (*p* < 0.001), and the mean duration of unstable resting sessions was significantly shorter (*p* < 0.001) in the P2 group. The duration for feeding was significantly longer (*p* = 0.014) in P1 compared to P0. Total activity per hour was significantly lower in P2 compared to P0 (*p* = 0.032) (Table 3).

**Figure 4:**
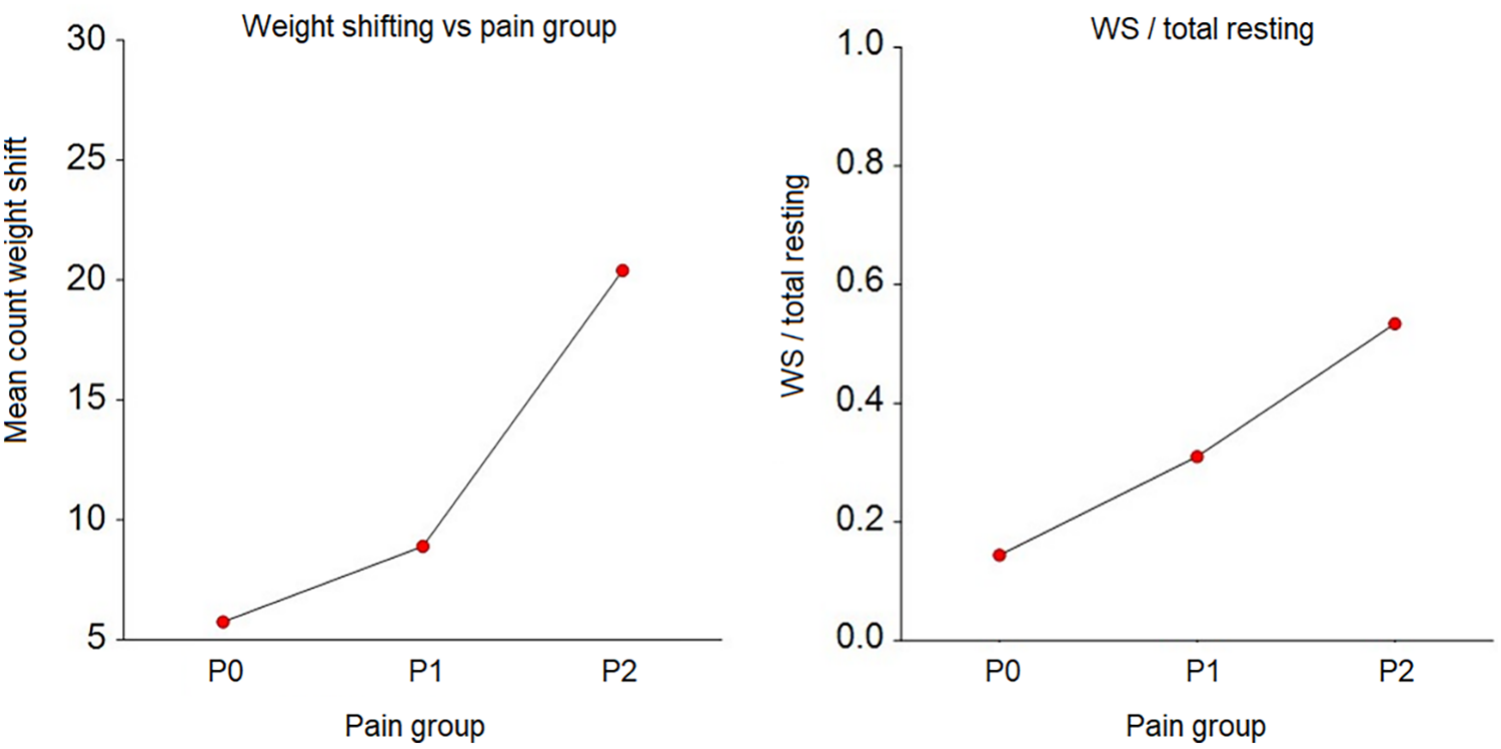
Frequency of weight shifting and weight shifting (WS)/total rest by pain group. P0 – pain free group, *n* = 8; P1 – group with mild to moderate pain, *n* = 10; P2 – group with severe pain, *n* = 2. The grouping is done based on the Pain Score Vetmeduni Vienna.

The frequency of weight shifting per hour (*p* < 0.001) and the ratio of weight shifting/total rest were significantly (*p* < 0.001 for WS/TRT) lower in P0 compared to P1 and P2 (see Figure 4, and Table 3).

## 4 Discussion

Evaluating equine pain in is a complex but indispensable aspect of effective clinical decision-making. The inherent subjectivity of pain behavior evaluation hinders objective and quantitative assessment. Thus, the integration of artificial intelligence (AI)-based analysis of video or sensor data emerges as a promising avenue. This technology offers the potential of continuous pain assessment over extended durations, minimizing observer bias and interference, thereby enhancing the precision and objectivity of pain evaluation in equine patients. However, successful implementation of AI-based analysis hinges on the identification and definition of robust, quantifiable parameters that can be readily analyzed based on video data and can accurately distinguish between painful and pain-free animals.

In this study, weight shifting, and unstable resting emerged as promising indicators for distinguishing between horses experiencing pain and those that are pain-free. In addition, severely painful horses (P2 group) exhibited lower frequencies of feeding and resting standing per hour compared to pain-free horses, while displaying a higher frequency of unstable resting per hour.

These findings align with previous research emphasizing postural behavior as a reliable indicator of pain, particularly in orthopedic conditions (14). Horses often redistribute weight away from a painful limb in search of relief, a behavior documented in various painful conditions such as laminitis (15). While healthy horses typically alternate weight-bearing on their hindlimbs during periods of rest, those experiencing pain may exhibit weight-shifting or adopt a three-legged body support (16–19).

Although previous studies have suggested thresholds for weight shifting indicative of physical fatigue, a definitive cut-off distinguishing physiological weight shifting from pain-related weight shifting remains elusive. While a frequency exceeding 7 weight shifts per 5 min has been linked to physical fatigue (20), horses with laminitis have been observed to shift weight between contralateral limbs up to 46 times per 10 min before analgesic intervention (21). In our study, we found a significantly higher incidence of weight shifting in painful horses (median: 13/h, range: 1–67) compared to pain-free counterparts (median: 2/h, range: 1–86). Notably, weight shifting was not only associated with orthopedic pain but was also observed in horses recovering from colic surgery that showed no clinical signs of laminitis. None of these horses underwent an orthopedic examination so we cannot exclude and subclinical orthopedic problem which was not mentioned by the owner or in the medical history. This unexpected finding invites further studies looking into the occurrence of weight shifting in a larger group of horses suffering from non-orthopaedic pain.

The significant difference in weight shifting frequency between horses before (median: 4/h, range: 1–56) and after (median: 3/h, range: 1–106) pain medication and the immediate decrease in weight shifting after administration of analgesia support the utility of weight shifting as an indicator for discomfort and pain assessment. Since horses primarily shift weight during rest, we calculated the weight-bearing ratio not only per hour but also relative to total rest time. This analysis revealed similarly significant differences between pain free horses (P0) and horses suffering from moderate to severe pain (P1, P2).

Based on the observation that some stationary horses exhibited movements beyond weight-shifting, including head and whole-body adjustments, we categorized stationary non-feeding time periods into two distinct behaviors: resting standing and unstable resting. Restlessness or fidgeting in horses has been suggested in previous research as a possible sign of discomfort (3). In this study, horses in pain tended to exhibit longer total resting periods disrupted frequently by short periods of unstable resting. However, a limitation of this study is the lack of differentiation between unstable resting and standing alert during environmental observation. These behaviors can resemble each other visually, potentially leading to misclassification. Therefore, while unstable resting shows promise as an indicator, further data are required for complete validation.

Animals experiencing pain or stress might exhibit behaviors such as avoiding stimuli, withdrawing, or becoming inactive (22). Behavioral variability, which refers to how frequently an animal transitions between different behaviors, has been recognized as an adaptive strategy indicative of exploratory behavior and overall good health. Various studies have explored behavior switching in the context of equine stereotypies, knowledge acquisition, anxiety, and food-related behaviors (23). The frequency of behavior switches provides insight into the responsiveness of an animal's disposition to internal and external stimuli. A reduction in behavior switching was linked to higher levels of pain due to joint inflammation (6). Our study showed similar results, with a significant reduction in activity in horses suffering from severe pain (P2). While the frequency of unstable resting per hour was higher in in P2 horses compared to pain-free (P0) horses, the number of resting standing episodes per hour was lower. Horses experiencing severe pain also exhibited a decrease in both feeding time and feeding attempts. However, the duration of individual feeding phases was significantly longer compared to pain-free horses. Nevertheless, further studies with larger sample sizes are needed to assess the utility of the time budget for feeding as an indicator of the severity of pain or discomfort.

The study has several limitations. The time-intensive nature of analyzing 24-h behavior restricted us to manual labeling of specific behaviors by a single observer during video observation. This approach introduces subjectivity and potential bias based on human perception.

Another limitation is the uneven distribution of data due to random horse selection regardless of existing problems. For ground truth data collection for the development of an AI model, horses were video recorded irrespective of their clinical condition. Analysis occurred retrospectively, after the horses were already discharged from the hospital, by observers blinded to the horses' medical history and treatments. Clinical decisions and treatments, including the administration of pain medication as deemed necessary, were provided according to each horse's specific medical condition, determined solely by the clinician's discretion, and were not influenced by the study. Other limitations of the study include the low number of horses experiencing severe pain, and the high individual variability among horses.

## 5 Conclusion

In conclusion, our study suggests that weight shifting and unstable resting, alongside the time budgets for feeding and total resting, seem to be promising indicators for distinguishing pain in horses. Weight shifting was significantly different between pain groups and could differentiate between mild-moderate pain (P1, P2) and pain-free (P0) conditions. It also showed significant differences in horses before and after receiving pain medication, indicating its potential utility in evaluating analgesic efficacy. Additionally, the duration and frequency of specific behavior sequences, like feeding, resting standing and unstable resting emerged as novel markers of equine pain that warrant further investigation. These parameters exhibited significant differences between pain groups (P2 group to P0 for all abovementioned, except for mean duration for feeding, where P1 was significantly different to P0, P2), indicating potential opportunities for AI-based analysis and automated pain assessment in equine medicine. However, while these indicators could differentiate between pain free and painful horses, they could not distinguish between different levels of pain experienced by the horses. Therefore, further studies with a larger study population, focusing on specific types of pain and pain intensities, with or without treatment, are needed to refine these findings. Leveraging (AI)-based analysis of video or sensor data based on the quantifiable indicators identified in this study may ultimately enhance pain assessment and management in horses, leading to improved welfare and clinical decision-making in equine medicine.

## Data Availability

The original contributions presented in the study are included in the article/Supplementary Material; further inquiries can be directed to the corresponding author/s.
