# Supplementary material for: Time budgets and weight shifting as indicators of pain in hospitalized horses
Source: Front Pain Res (Lausanne). 2024 Jul 23;5:1410302. doi: 10.3389/fpain.2024.1410302 (PMC11300370; doi:10.3389/fpain.2024.1410302)
Supplement: Supplementary file 1 [file Table1.docx]

Suppl. Table 1: Overview of time budgets for each horse. Values are provided as median (range) per hour over 24h. P0 – pain free group; P1 – group with mild to moderate pain; P2 – group with severe pain.

| **Horse** | **Pain group** | **Feeding %** | **Resting standing %** | **Unstable resting %** | **Total Resting %** | **Lying %** | **Moving %** |
| --- | --- | --- | --- | --- | --- | --- | --- |
| A | P1 | 19 (0-100) | 4 (0-94) | 34 (0-100) | 78 (0-100) | 0 | 0 (0-2.3) |
| B | P0 | 46 (0-99) | 5 (0-95) | 4 (0-95) | 30 (0-96) | 0 (9-100) | 0 (0-3.6) |
| C | P1 | 42 (0-96) | 10.6 (0-46) | 31 (3-98) | 56 (4-98) | 0 | 1,6 (0-7.5) |
| D | P1 | 43 (0.4-100) | 8.6 (0-56.3) | 44 (0-70) | 57 (0-100) | 0 | 0 (0-8) |
| E | P0 | 52 (2.4-100) | 7.2 (0-52) | 7.2 (0-52.3) | 48 (0-98) | 0 | 0 (0-2) |
| F | P0 | 28 (0.7-82) | 29 (0-84) | 18 (10-66.37) | 71 (18-98) | 0 (0-15) | 0.8 (0.2-2.4) |
| G | P0 | 50 (0-93) | 0 (0-42) | 39 (7-90) | 49 (6,8-100) | 0 (0-5) | 0.5 (0-8.3) |
| H | P0 | 53 (0-88) | 16 (0-90) | 14 (0-31) | 35 (9-100) | 0 (0-91) | 1.5 (0-8) |
| I | P1 | 51 (0-98) | 9 (0-65) | 22 (0-84) | 47 (0-100) | 0 | 2 (0-20) |
| J | P0 | 41 (0.3-93) | 20 (0-67) | 24 (0-42) | 42 (6,7-92) | 0 (0-55) | 0 (0-6) |
| K | P1 | 36 (0-77) | 38 (0-77) | 31 (1-88) | 62 (23-99) | 0 | 1 (0-13) |
| L | P1 | 56 (1-98) | 29 (0-88) | 11 (2-30) | 44 (2-100) | 0 | 0 (0-1.6) |
| M | P1 | 60 (0-100) | 12 (0-98) | 4 (0-51) | 34 (0-100) | 0 (0-97) | 1 (0-3) |
| N | P1 | 87 (0.4-100) | 2 (0-66) | 6,4 (0-56) | 13 (0-99) | 0 | 0 (0-2) |
| O | P1 | 29 (0-92) | 14 (0-94) | 22 (0-97) | 52 (6-98) | 0 (0-94) | 1 (0-2) |
| P | P1 | 30 (0-87) | 10 (0-65) | 29 (0-75) | 58 (0-100) | 0 (0-99) | 1 (0-4) |
| Q | P2 | 24 (0-76) | 41 (0-86) | 22 (0-100) | 67 (0-100) | 0 (0-79) | 0 (0-4) |
| R | P0 | 54 (0-100) | 15 (0-85) | 13 (0-74) | 43 (0-94) | 0 (0-43) | 1.3 (0-11.2) |
| S | P0 | 33 (0-89) | 24 (0-79) | 19 (2-64) | 57 (11-97) | 0 (0-44) | 1 (0-16) |
| T | P2 | 48 (0-100) | 18 (0-89) | 14 (0-47) | 36 (0-100) | 0 | 2 (0-37) |
